# Survival Comparison Between Squamous Cell Carcinoma and Adenocarcinoma for Radiotherapy-Treated Patients with Stage IIB-IVA Cervical Cancer

**DOI:** 10.3389/fonc.2022.895122

**Published:** 2022-07-22

**Authors:** Fangjie Chen, Long Chen, Yu Zhang, Lei Shi, Hong’en Xu, Tao Song

**Affiliations:** ^1^ Department of Outpatient Nursing, Zhejiang Provincial People’s Hospital, Affiliated People’s Hospital, Hangzhou Medical College, Hangzhou, China; ^2^ Graduate School, Zhejiang Chinese Medical University, Hangzhou, China; ^3^ Cancer Center, Department of Radiation Oncology, Zhejiang Provincial People’s Hospital, Affiliated People’s Hospital, Hangzhou Medical College, Hangzhou, China; ^4^ Department of Nursing (5-11 Ward), Zhejiang Provincial People’s Hospital, Affiliated People’s Hospital, Hangzhou Medical College, Hangzhou, China

**Keywords:** cervical cancer, squamous cell carcinoma, adenocarcinoma, SEER, survival

## Abstract

**Objective:**

To compare the prognostic significance of adenocarcinoma (AC) with squamous cell carcinoma (SCC) on overall survival (OS) in patients with stage IIB-IVA cervical cancer (CC) treated by external beam radiotherapy (EBRT) and brachytherapy (BRT) with/without chemotherapy registered in the Surveillance, Epidemiology, and End Results database.

**Methods:**

Data of eligible patients were extracted between 2004 and 2016. A univariate analysis was conducted using the cumulative incidence function (CIF) by considering competing events and compared using Gray’s test. The significant variables in univariate analysis were further evaluated in a multivariate analysis performed with the Fine-Gray regression model. Propensity score matching (PSM) analysis was also employed to reconfirm the results found in the present study.

**Results:**

A total of 2,243 patients with SCC and 176 patients with AC were extracted from the database. The 5-year OS rates were 57.8% in the SCC group and 52.8% in the AC group. 149 patients died of causes other than CC—considered as competing events. Compared with the SCC group, patients diagnosed with AC had statistically significant worse 5-year OS rate before and after PSM. In the multivariate Fine-Gray regression model, the histological subtype of AC was proven as an independent prognostic factor associated with poorer OS before [hazard ratio (HR) = 1.340; 95% confidence interval (CI): 1.081-1.660; *P* = 0.007] and after [HR = 1.376; 95% CI: 1.107-1.711; *P* = 0.004] PSM.

**Conclusions:**

The histological subtype of AC is significantly correlated with impaired OS as an independent prognostic variable in patients with stage IIB-IVA CC who received EBRT and BRT compared to patients with SCC. Future studies should incorporate effective and individualized treatment strategies into clinical decision-making to improve the unsatisfactory survival outcomes for patients with AC.

## Introduction

Globally, cervical cancer (CC) ranks as the fourth most frequently diagnosed neoplasm and the fourth leading cause of cancer deaths in females. Furthermore, CC has an incidence rate of 604,127 new cases and over 340,000 deaths in 2020 as reported by the GLOBOCAN study (1). The International Federation of Gynecology and Obstetrics (FIGO) states that stage IIB-IVA cancer accounts for more than 80% of all cases in patients with CC from low/middle-income countries. The standard treatment option worldwide consists of external beam radiotherapy (EBRT) with concurrent chemotherapy (CT) followed by brachytherapy (BRT), which provide a 5-year overall survival (OS) rate of ~60% (2, 3).

Squamous cell carcinoma (SCC) is the predominant type of CC accounting for 70–80% of all CCs (4). As a result of regular cervical cytological screening and other medical developments, the morbidity of SCC has decreased during the past decades. In contrast, it was observed that there has been a recent rise in the incidence of adenocarcinoma (AC) (5, 6). However, due to its rarity, few studies have proposed effective management strategies to address AC. An earlier matched case-control study in the USA compared the survival outcomes between SCC and AC among 162 patients with CC but found no significant difference in survival between the two histological subtypes when matching the tumor stages (7). In contrast, a 2015 retrospective study reported the survival outcomes of 29 patients with AC who received concurrent chemoradiotherapy (CCRT) compared to that of 39 patients with SCC (8). They found that the patients with AC were less responsive to CCRT and had poorer OS compared with patients with SCC (7.4 years vs 11.0 years, respectively). Despite the difference in OS, the current FIGO and National Comprehensive Cancer Network (NCCN) clinical practice guidelines still suggest similar treatment modalities for SCC and AC. Therefore, the question remains how to best manage these two different histological subtypes.

To explore this discrepancy, we aimed to compare the prognostic significance between SCC and AC in patients with stage IIB-IVA diseases who received both EBRT and BRT with/without CT using data from the Surveillance, Epidemiology, and End Results (SEER) database using competing-risks analysis and propensity score matching (PSM) analysis.

## Materials and Methods

### Ethical Statement

It was not necessary to get written informed consent for participating in the present research as the information contained in the SEER database has been de-identified and is publicly available following authorization. The present research was exempted from ethical assessment by the Institutional Review Board of Zhejiang Provincial People’s Hospital.

### Data Collection and Selection Criteria

**Figure 1:**
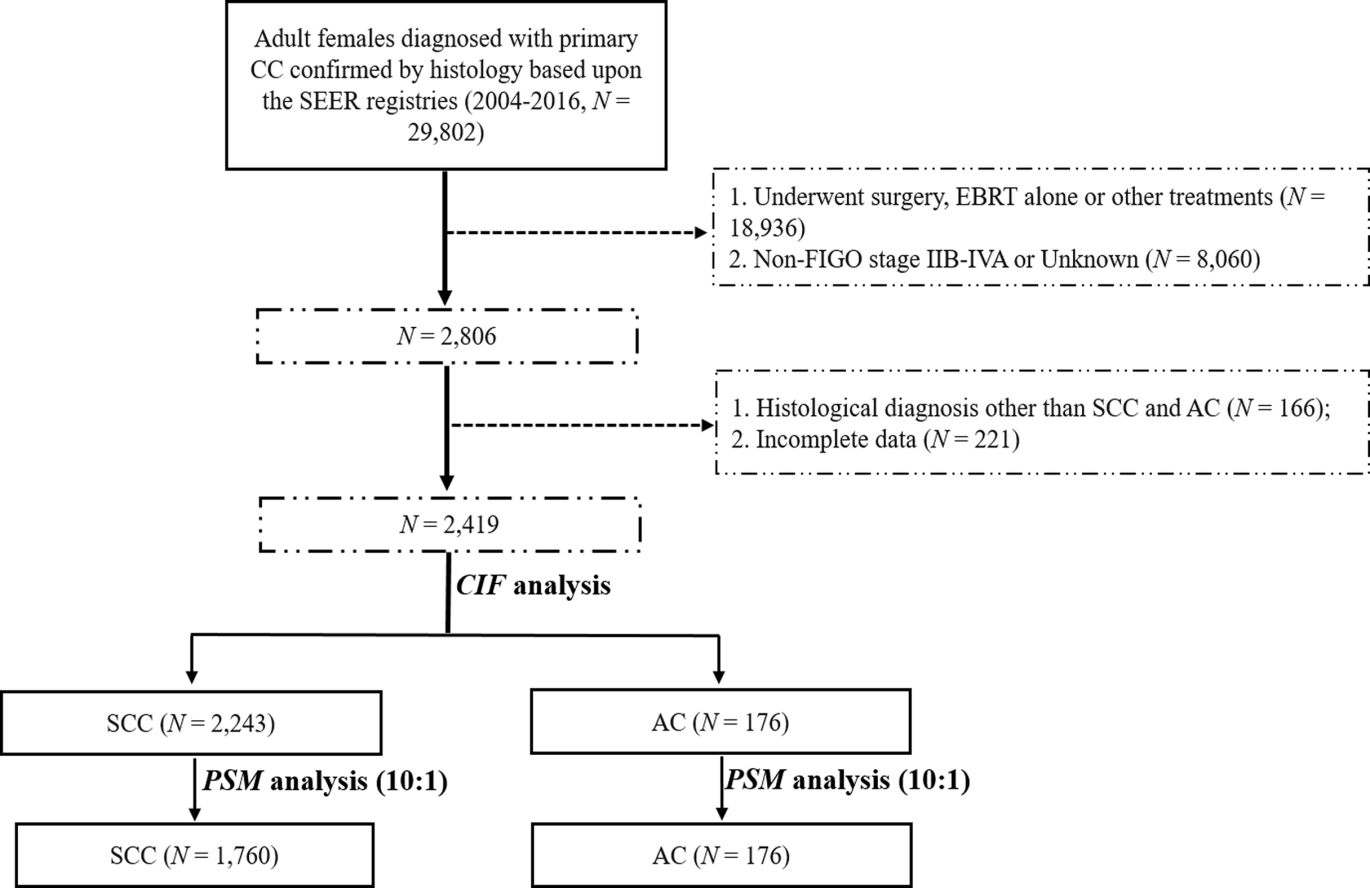
Patient selection flowchart.

We used SEER*Stat v8.3.6 (username: 10579-Nov2019) to retrieve the data of patients with CC from 2004 to 2016 under the International Classification of Diseases for Oncology, 3rd Edition (ICD-O-3): C53.8 (overlapping lesion of cervix uteri) and C53.9 (cervix uteri, unspecified). We included patients with CC according to the following inclusion criteria: 1) histopathologic diagnosis of SCC and AC; 2) primary diagnosis of stage IIB-IVA diseases based on the 2014 FIGO staging manual (9); and 3) a clear indication that patients received EBRT followed by BRT without surgery as indicated in the SEER program. The major exclusion criteria were: 1) patients who had more than one primary cancer; 2) patients aged less than 18 years old or underwent other treatment modalities as indicated in the registry; and 3) patients with ambiguous data or with unknown survival time. The selection flow chart is presented in **Figure 1**.

### Data Processing

Variables for each patient used in the current study included age at diagnosis, race, marital status, histology type, differentiation grade, the 2014 FIGO stage, tumor size, use of CT, as well as survival information, including survival status, survival time in months, cause of death (COD) and specific site recode, and cause-specific death classification. The last two variables indicate whether the person died of CC (tumor-specific death, TSD) or from causes other than CC (non-tumor-specific death, NTSD). We considered NTSDs as competing events in the present study. The 2014 FIGO staging manual was employed in the present study given that our previous study have demonstrated non-superior prognostic impact of the 2018 FIGO staging system over the 2014 FIGO staging system (10). The cut-off point for the age at diagnosis was set at 70-years old as adopted by other SEER studies (11, 12). Classification of the other variables was assorted according to our previous studies (13, 14).

### Statistical Analysis

OS was defined as the duration from the diagnosis of CC to the time of death caused by any reason or the last follow-up registered in the database. The baseline characteristics of patients were summarized by descriptive statistics and frequency tables. One-way analysis of variance (ANOVA) was used to compare the proportions of different groups. Survival curves for OS were estimated using the Kaplan-Meier method. To balance the enrolled patients and reduce the selection bias of baseline characteristics between the two groups, we performed a PSM analysis for covariates, including marital status, and the 2014 FIGO stage at a ratio of 1:10 for AC: SCC. The PSM analysis was conducted as described in our previous studies (14, 15). A univariate analysis was employed using the cumulative incidence function (CIF) to determine the probability of each covariate and Gray’s test to evaluate the difference between groups. Factors with a *P* value less than 0.05 in the univariate analysis were selected and entered into a multivariate competing-risks survival analysis *via* the proportional subdistribution hazard model by the Fine-Gray test (16). Hazard ratios (HRs) and their 95% confidence intervals (CIs) were also calculated in the analysis. The probability of the two-sided *P* values less than 0.05 were considered statistically significant.

Statistical analyses were performed with the Statistical Package for the Social Sciences (SPSS) v25.0 software (IBM Corporation, Armonk, NY, USA) and R v3.6.2 software (https://www.r-project.org, Institute for Statistics and Mathematics, Vienna, Austria) with the R “*MatchIt, survival, and cmprsk*” packages.

## Results

### Demographic and Baseline Characteristics

A total of 2,419 patients with stage IIB-IVA diseases who received EBRT followed by BRT with or without CT from 2004 to 2016 were retrieved from the SEER registry. It is worth pointing out that the median size of known masses was 60mm with only 168 (7.5%) patients had tumors less than 40mm among all patients. The enrolled patients were then divided into two groups based on their histological subtypes: the SCC group (*N* = 2,243) and AC group (*N* = 176). The baseline characteristics of these two patient groups are demonstrated in **Table 1**. Given the significant differences in marital status (*P* = 0.001) and 2014 FIGO stage (*P* = 0.005) between the SCC and AC groups, a PSM analysis was performed at a ratio of 1:10 for AC: SCC to retain an approximate statistical power and balance the distribution of these baseline characteristics. After matching, no demographic, clinical, and treatment characteristics were significantly different between the two groups.

**Table 1:** Baseline characteristics of patients with stage IIB-IVA cervix carcinoma who received EBRT and BRT.

Characteristic	Before PSM			After PSM		
	SCC (n, %)	AC (n, %)	*P* value	SCC (n, %)	AC (n, %)	*P* value
*Age at diagnosis*			0.338			0.195
<70	1979 (88.2)	151 (85.8)		1567 (89.0)	151 (85.8)	
≥70	264 (11.8)	25 (14.2)		193 (11.0)	25 (14.2)	
*Marital status*			0.001			0.499
Married	833 (37.1)	88 (50.0)		833 (47.3)	88 (50.0)	
Unmarried and others	1410 (62.9)	88 (50.0)		927 (52.7)	88 (50.0)	
*Race*			0.473			0.662
White	1613 (71.9)	131 (74.4)		1283 (72.9)	131 (74.4)	
Non-White	630 (28.1)	45 (25.6)		477 (27.1)	45 (25.6)	
*Differentiation*			0.353			0.349
Well or fairly	851 (37.9)	62 (35.2)		675 (38.4)	62 (35.2)	
Poorly or undifferentiated	779 (34.7)	57 (32.4)		605 (34.4)	57 (32.4)	
Unknown	613 (27.4)	57 (32.4)		480 (27.2)	57 (32.4)	
*2014 FIGO stage*			0.005			0.158
IIB	1238 (55.2)	104 (59.1)		1010 (57.4)	104 (59.1)	
IIIA	137 (6.1)	21 (11.9)		137 (7.8)	21 (11.9)	
IIIB	775 (34.6)	46 (26.1)		563 (32.0)	46 (26.1)	
IVA	93 (4.1)	5 (2.9)		50 (2.8)	5 (2.9)	
*Tumor size (mm)*			0.725			0.665
<60	668 (29.8)	50 (28.4)		536 (30.5)	50 (28.4)	
≥60	887 (39.5)	75 (42.6)		689 (39.1)	75 (42.6)	
Unknown	688 (30.7)	51 (29.0)		535 (30.4)	51 (29.0)	
*Chemotherapy*			0.960			0.889
No/Unknown	168 (7.5)	13 (7.4)		125 (7.1)	13 (7.4)	
Yes	2075 (92.5)	163 (92.6)		1635 (92.9)	163 (92.6)	

PSM, Propensity score-matching; SCC, squamous cell carcinoma; AC, adenocarcinoma; FIGO, International Federation of Gynecology and Obstetrics.

### Survival Results Before and After PSM

Before matching, the 3- and 5-year OS rates in the whole cohort were 65.0% (95% CI 0.628–0.672) and 57.6% (95% CI 0.552–0.600), respectively, with a median OS time of 110.0 months (95% CI not reached). Of the 2,419 patients, 921 deaths were registered in the database with 837 deaths in the SCC group and 84 events in the AC group. A significant risk of death was observed in the AC group (*P* = 0.006). Among the 921 deaths, a total of 149 (16.2%) patients with CC died of NTSDs: 138 patients in the SCC group and 11 patients in the AC group. Of the 149 patients that died of NTSDs, 60 (40.3%) patients died of other causes or data not available in the SEER registry. Heart diseases (28, 18.8%), accidents and adverse effects (10, 6.7%), and septicemia (10, 6.7%) ranked second to fourth for NTSDs (**Table S1**). There was no significant difference in NTSDs between the two histological subtypes (*P* = 0.421). The 3- and 5-year OS rates in the SCC group were 65.0% (95% CI 0.628–0.672) and 57.8% (95% CI 0.554–0.602), respectively, with a median OS time of 123.0 months (95% CI not reached). The 3- and 5-year OS rates in the AC group were 64.4% (95% CI 0.568–0.720) and 52.8% (95% CI 0.442–0.614), respectively, with a median OS time of 71.0 months (95% CI 51.3–90.7). After matching, the 3- and 5-year OS rates in the SCC group were 66.3% (95% CI 0.639–0.687) and 59.1% (95% CI 0.566–0.616), respectively. The corresponding 3- and 5-year OS rates in the AC group were 64.4% (95% CI 0.568–0.720) and 52.8% (95% CI 0.442–0.614), respectively (**Figure S1**).

### Fine-Gray Regression Model Before and After PSM

**Figure 2:**
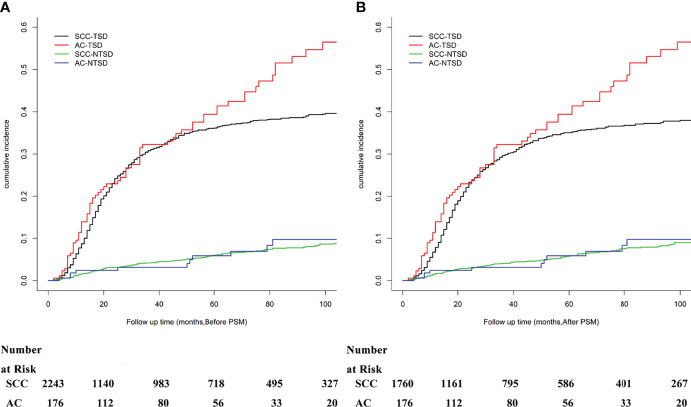
The cumulative risk curves according to TSD and NTSD before **(A)** and after **(B)** PSM.

Except for the significant differences between the histological subtype (SCC vs AC, *P* = 0.012), a univariate analysis using Grey’s test indicated that tumor differentiation (*P* = 0.015), 2014 FIGO stage (*P* < 0.001), and tumor size (*P* < 0.001) significantly correlated with the prognosis of patients with stage IIB-IVA CC. The cumulative risk curves for SCC and AC before and after PSM are shown in **Figure 2**. There was no significant difference of NTSD between two histological groups. The CIF values increased after 36 months and were higher for the following eight variables: patients aged less than 70 years old, unmarried and others, of the white race, with AC subtype, showing poorly or undifferentiated tumor grade, at an advanced FIGO stage, large tumor size, and having no or unknown status of receiving CT. The CIF values among patients with SCC were 30.8% and 36.2% at 36 and 60 months, respectively. The corresponding figures for the patients with AC were 32.3%, and 39.4%, respectively. After matching, the histological subtype (*P* = 0.004), 2014 FIGO stage (*P* < 0.001), and tumor size (*P* < 0.001) were all significant variables that impacted the prognosis of patients with stage IIB-IVA CC. The results from the univariate analysis and CIF values are presented in **Table 2**.

**Table 2:** Univariate analysis of OS using a competing risk model in patients with stage IIB-IVA CC who received EBRT and BRT before and after PSM.

Factors	Gray’s test	*P* value	CIF before PSM		Gray’s test	*P* value	CIF after PSM	
			36-months	60-months			36-months	60-months
*Age (years)*	0.001	0.970			0.004	0.948		
< 70			0.314	0.365			0.302	0.355
≥ 70			0.275	0.352			0.273	0.346
*Marital status*	3.760	0.052			1.329	0.249		
Married			0.287	0.344			0.287	0.344
Unmarried and others			0.323	0.376			0.310	0.364
*Race*	1.529	0.216			1.503	0.220		
White			0.318	0.373			0.308	0.363
Non-white			0.288	0.341			0.273	0.332
*Histology*	6.248	0.012			8.296	0.004		
SCC			0.308	0.362			0.296	0.350
AC			0.323	0.394			0.323	0.394
*Differentiation*	8.345	0.015			5.836	0.054		
Well or fairly			0.303	0.350			0.294	0.346
Poorly or undifferentiated			0.338	0.400			0.324	0.388
Unknown			0.282	0.340			0.274	0.324
*2014 FIGO stage*	101.587	< 0.001			75.354	< 0.001		
IIB			0.222	0.279			0.216	0.276
IIIA			0.335	0.418			0.335	0.418
IIIB			0.419	0.466			0.422	0.466
IVA			0.500	0.549			0.434	0.471
*Tumor size (mm)*	25.857	< 0.001			16.509	< 0.001		
< 60			0.229	0.293			0.225	0.293
≥ 60			0.352	0.405			0.339	0.390
Unknown			0.329	0.379			0.318	0.370
*Chemotherapy*	1.376	0.241			1.418	0.234		
No/unknown			0.356	0.403			0.365	0.407
Yes			0.306	0.361			0.294	0.351

CIF, cumulative incidence function.

We next entered the three variables that were statistically significant in the univariate analysis before and after PSM (histological subtype, 2014 FIGO stage, and tumor size) and the one factor (tumor differentiation before PSM) into a Fine-Gray regression model. The Fine-Gray regression model before PSM indicated that the following parameters were all independent prognostic factors: histological subtype (SCC vs AC, *P* = 0.007, HR = 1.340, 95% CI 1.081–1.660), tumor differentiation (well or fairly differentiated vs poorly or undifferentiated, *P* < 0.001, HR = 1.293, 95% CI 1.113–1.502), 2014 FIGO stage (IIB vs IIIA, *P* < 0.001, HR = 1.543, 95% CI 1.196–1.990; IIB vs IIIB, *P* < 0.001, HR = 1.864, 95% CI 1.622–2.142 and IIB vs IVA, *P* < 0.001, HR = 2.657, 95% CI 1.952–3.618), and tumor size (< 60 vs ≥ 60, *P* = 0.019, HR = 1.222, 95% CI 1.034–1.444; < 60 vs Unknown, *P* = 0.006, HR = 1.270, 95% CI 1.070–1.508). After matching, the histological subtype (SCC vs AC, *P* = 0.004, HR = 1.376, 95% CI 1.107–1.711) remained as an independent prognostic variable associated with OS (**Table 3**).

**Table 3:** Multivariate analysis of OS in patients with CC using the Fine-Gray regression model before and after PSM.

Factor	CIF before PSM				CIF after PSM			
	P value	HR	95% CI	95% CI	P value	HR	95% CI	95% CI
			Lower	Upper			Lower	Upper
*Histology, SCC vs. AC*	0.007	1.340	1.081	1.660	0.004	1.376	1.107	1.711
*Differentiation, reference: Well or fairly*	1.000				–			
Poorly or undifferentiated	< 0.001	1.293	1.113	1.502				
Unknown	0.725	1.031	0.870	1.221				
*2014 FIGO stage, reference: IIB*	1.000				1.000			
IIIA	< 0.001	1.543	1.196	1.990	< 0.001	1.576	1.215	2.043
IIIB	< 0.001	1.864	1.622	2.142	< 0.001	1.953	1.669	2.285
IVA	< 0.001	2.657	1.952	3.618	< 0.001	2.259	1.454	3.510
*Tumor size (mm), reference: < 60*	1.000				1.000			
≥ 60	0.019	1.222	1.034	1.444	0.071	1.185	0.986	1.425
Unknown	0.006	1.270	1.070	1.508	0.077	1.189	0.981	1.441

PSM, Propensity score-matching; HR, Hazard ratio; CI, confidence interval; SCC, squamous cell carcinoma; AC, adenocarcinoma; FIGO, International Federation of Gynecology and Obstetrics.

## Discussion

Since the influence of cervical AC on survival remains controversial for patients with stage IIB-IVA CC who received EBRT and BRT, the findings in the present study add to a growing body of literature indicating that patients with CC and concurrent AC had significantly decreased OS times when compared with the survival outcomes of patients with SCC before and after PSM analysis. Furthermore, we demonstrated that the histological subtype of AC was an independent prognostic factor that significantly decreased OS in the Fine-Gray regression model.

Although both malignancies originate from the cervix of the uterine, heterogeneities exist between SCC and AC in many respects including anatomic origin, biological behavior, rate of lymph node and/or distant metastasis, as well as sensitivity to radiotherapy (RT) and CT (17, 18). Except for the aforementioned small sample study (8), a Thailand report retrospectively compared treatment outcomes between SCC and AC at a ratio of 2:1 in patients with locally advanced CC (19). Among 423 enrolled patients, those with the histological subtype AC were observed to have a significant reduction in achieving a complete response (CR; 94.7% for SCC and 86.5% for AC) and a significantly prolonged time to reach a CR after receiving RT/CCRT. However, these clinical indicators did not translate to significant benefit in the 5-year OS rates for patients with SCC and AC, which potentially resulted from the enrollment of patients without FIGO stage IIIA in their study. It should be pointed out that the significant OS difference between SCC and AC was mainly contributed after 5 years in the current study, which also need further observation. On the contrary, another large sample, retrospective study compared the efficiency of RT/CCRT between SCC and AC among 815 patients with stage IB-IVA, of whom 71 patients were diagnosed with AC (20). Using multivariate Cox regression analysis, the study demonstrated AC as an independent prognostic factor associated with decreased OS before and after PSM, regardless of whether the treatment delivery consisted of RT alone or CCRT. Similar findings were also reported in other retrospective studies (21, 22), whereby patients with AC/adenosquamous carcinoma (ASC) were observed to suffer significant reduction of 5-year disease-free survival, locoregional failure-free survival, and distant metastasis-free survival before and after PSM. The authors then concluded that patients with the histological subtypes AC/ASC should receive more effective and aggressive treatments to overcome this survival disparity.

One strategy to improve the dismal survival results among AC patients is using new cytotoxic drugs like paclitaxel (23). In a large sample, randomized controlled trial from China (24), 880 patients with FIGO stages IIB-IVA diseases and AC subtype were allocated to receive either standard CCRT with cisplatin (group A) or CCRT with one cycle of neoadjuvant CT plus two cycles of consolidation CT with paclitaxel and cisplatin after CCRT (group B). They found that the 5-year local-regional recurrence-free survival rates were significantly different between groups A and B (62.9% vs 74.7%, respectively). Another useful technique to improve the survival benefits for patients with AC is using modern RT, such as carbon-ion RT. In a PSM study in Japan (25), the authors compared the survival outcomes for patients with locally advanced AC who underwent either carbon-ion RT alone or carbon-ion RT and CT. A total of 26 patients with AC per group was analyzed and the 5-year OS rate was demonstrated to be significantly better in the carbon-ion RT and CT group (72%) than in the RT alone group (46%) with acceptable treatment-related sequelae.

In our study, we employed CIF analysis and the Fine-Gray regression model in our statistical analysis based on the following considerations: the 5-year OS rate for patients with stage IIB-IVA CC was ~60.0%, and 149 (16.2%) patients were recorded to have COD other than CC in the SEER database, which was considered as competing risks. It should be noted that, to the best of our knowledge, almost all of the studies in the literature which confirmed the prognostic impact of AC on survival outcomes was based on the results suggested by Cox proportional hazard regression models (26). The relatively long-term survival and the non-homogeneity in the CODs might cause bias in calculating the real impact of the histological subtype on survival outcomes. In this regard, Yang et al. previously compared the Fine-Gray regression model with the Cox regression model in penile cancers also with data extracted from the SEER database (27). Their results indicated that the Fine-Gray regression model could estimate the cumulative incidence of death and the effect of variables on the hazard rate more accurately than the Kaplan-Meier and Cox regression models. Similar findings were also reported by other researchers (28, 29), and using the Fine-Gray regression model might avoid the overestimation of survival outcomes in the presence of a competing risk.

This study is not without limitations. First, this study is a retrospective study. Second, some important data, including the performance status, the status of human papilloma virus infection, pathological subtypes based on the updated International Endocervical Adenocarcinoma Criteria and Classification (30), hemoglobin levels, and treatment failure pattern (local-regional or distant metastasis) were not available in the SEER database during the study timeframe. Third, with the progress of anti-cancer therapies in this field, treatment-related parameters, like EBRT modalities and dosages, newer CT regimens and cycles, low/high-dose-rate BRT, immunotherapies, toxicities, overall treatment time and subsequent treatment combinations are also unknown, which might certainly lead to a bias in the final inferences. Finally, the results in the current study were explored based on data registered only in the SEER database, whether the findings generated in the current study apply to other populations, such as that of other industrialized countries or low/middle-income areas, needs to be confirmed in the future.

## Conclusions

The present study aimed to compare the impact of the histological subtype of AC with SCC on the OS outcomes among patients with stage IIB-IVA CC who received EBRT and BRT registered in the SEER database from 2004 to 2016. By comparing data before and after PSM analysis with the application of CIF analysis and the Fine-Gray regression model, AC remained an independent prognostic factor for poorer OS outcomes. Therefore, when treating patients with stage IIB-IVA CC and AC, more aggressive and individualized treatment options should be considered to improve the unsatisfactory survival outcomes of patients with AC.

## Data Availability Statement

The original contributions presented in the study are included in the article/**Supplementary Material**. Further inquiries can be directed to the corresponding authors.

## Ethics Statement

Ethical review and approval were not required for the study on human participants in accordance with the local legislation and institutional requirements. Written informed consent for participation was not required for this study in accordance with the national legislation and the institutional requirements.

## Author Contributions

FC: conception and design, drafting, final approval, and accountable for aspects; LC: provision of study materials, collection and assembly of data, drafting, final approval, and accountable for aspects; YZ: provision of study materials, collection and assembly of data, drafting, final approval, and accountable for aspects; LS: data analysis and interpretation, drafting, final approval, and accountable for aspects; HX: conception and design, provision of study materials, collection and assembly of data, drafting, final approval, accountable for aspects; TS: conception and design, provision of study materials, collection and assembly of data, drafting, final approval, and accountable for aspects. All authors contributed to the article and approved the submitted version.

## Conflict of Interest

The authors declare that the research was conducted in the absence of any commercial or financial relationships that could be construed as a potential conflict of interest.

## Publisher’s Note

All claims expressed in this article are solely those of the authors and do not necessarily represent those of their affiliated organizations, or those of the publisher, the editors and the reviewers. Any product that may be evaluated in this article, or claim that may be made by its manufacturer, is not guaranteed or endorsed by the publisher.

## Supplementary Material

Supplementary Figure 1 Overall survival of the entire cohort, SCC group, and AC group before **(A)** and after **(B)** PSM.

Supplementary Table 1 Causes of death (COD) other than CC registered in the SEER database.
