# Supplementary figures and images for: Survival Comparison Between Squamous Cell Carcinoma and Adenocarcinoma for Radiotherapy-Treated Patients with Stage IIB-IVA Cervical Cancer
Source: Front Oncol. 2022 Jul 22;12:895122. doi: 10.3389/fonc.2022.895122 (PMC9352995; doi:10.3389/fonc.2022.895122)

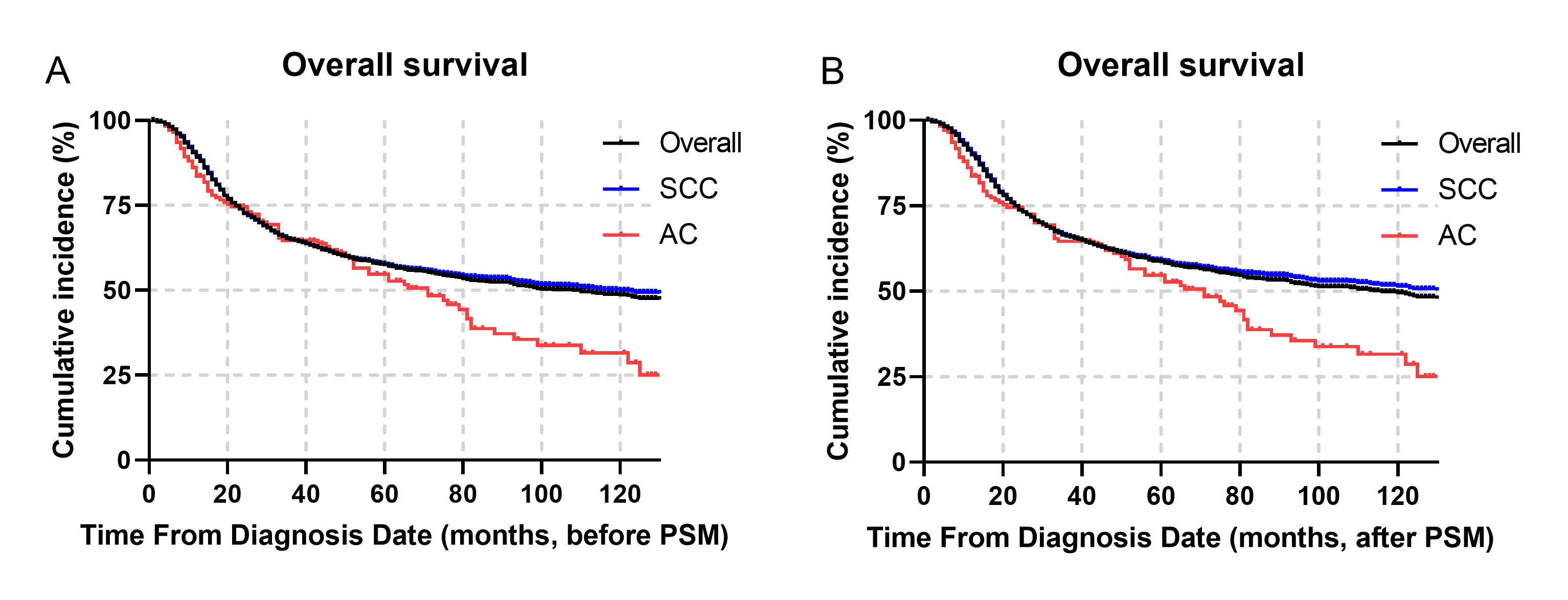

Supplement: Supplementary Figure 1 — Overall survival of the entire cohort, SCC group, and AC group before (A) and after (B) PSM. [file Image_1.tif]
