# Supplementary material for: Survival Comparison Between Squamous Cell Carcinoma and Adenocarcinoma for Radiotherapy-Treated Patients with Stage IIB-IVA Cervical Cancer
Source: Front Oncol. 2022 Jul 22;12:895122. doi: 10.3389/fonc.2022.895122 (PMC9352995; doi:10.3389/fonc.2022.895122)
Supplement: Supplementary Table 1 — Causes of death (COD) other than CC registered in the SEER database. [file Table_1.docx]

Table S1. Causes of death (COD) other than CC registered in the SEER database.

| **COD other than CC** | *N*, (%) |
| --- | --- |
| Other Cause of Death or unknown | 60 (40.3) |
| Diseases of Heart | 28 (18.8) |
| Accidents and Adverse Effects | 10 (6.7) |
| Septicemia | 10 (6.7) |
| Chronic Obstructive Pulmonary Disease and Allied Cond | 8 (5.4) |
| Nephritis, Nephrotic Syndrome and Nephrosis | 8 (5.4) |
| Diabetes Mellitus | 5 (3.3) |
| Other Infectious and Parasitic Diseases including HIV | 5 (3.3) |
| Cerebrovascular Diseases | 4 (2.6) |
| Congenital Anomalies | 2 (1.3) |
| Other Diseases of Arteries, Arterioles, Capillaries | 2 (1.3) |
| Alzheimers | 1 (0.7) |
| Aortic Aneurysm and Dissection | 1 (0.7) |
| Complications of Pregnancy, Childbirth, Puerperium | 1 (0.7) |
| Chronic Liver Disease and Cirrhosis | 1 (0.7) |
| Hypertension without Heart Disease | 1 (0.7) |
| Pneumonia and Influenza | 1 (0.7) |
| Stomach and Duodenal Ulcers | 1 (0.7) |
